# Dense Plasma Focus-Based Nanofabrication of III–V Semiconductors: Unique Features and Recent Advances

**DOI:** 10.3390/nano6010004

**Published:** 2015-12-29

**Authors:** Onkar Mangla, Savita Roy, Kostya (Ken) Ostrikov

**Affiliations:** 1 Department of Physics and Astrophysics, University of Delhi, Delhi 110007, India; onkarmangla@gmail.com; 2 Physics Department, Hindu College, University of Delhi, Delhi 110007, India; 3 Physics Department, Daulat Ram College, University of Delhi, Delhi 110007, India; 4 School of Chemistry, Physics and Mechanical Engineering, Queensland University of Technology (QUT), Brisbane 4000, Australia; kostya.ostrikov@csiro.au; 5 Plasma Nanoscience Laboratories, Commonwealth Scientific and Industrial Research Organisation, P.O. Box 218, Lindfield 2070, Australia; 6 Plasma Nanoscience, School of Physics, The University of Sydney, Sydney 2006, Australia

**Keywords:** III–V semiconductors, nanofabrication, dense plasma focus, rapid thermal annealing, photoluminescence, transmittance

## Abstract

The hot and dense plasma formed in modified dense plasma focus (DPF) device has been used worldwide for the nanofabrication of several materials. In this paper, we summarize the fabrication of III–V semiconductor nanostructures using the high fluence material ions produced by hot, dense and extremely non-equilibrium plasma generated in a modified DPF device. In addition, we present the recent results on the fabrication of porous nano-gallium arsenide (GaAs). The details of morphological, structural and optical properties of the fabricated nano-GaAs are provided. The effect of rapid thermal annealing on the above properties of porous nano-GaAs is studied. The study reveals that it is possible to tailor the size of pores with annealing temperature. The optical properties of these porous nano-GaAs also confirm the possibility to tailor the pore sizes upon annealing. Possible applications of the fabricated and subsequently annealed porous nano-GaAs in transmission-type photo-cathodes and visible optoelectronic devices are discussed. These results suggest that the modified DPF is an effective tool for nanofabrication of continuous and porous III–V semiconductor nanomaterials. Further opportunities for using the modified DPF device for the fabrication of novel nanostructures are discussed as well.

## 1. Introduction

Nanostructured materials have gained strong recent interest due to their superior properties as compared to their thin films and bulk counter parts. These properties of nanostructures result in several advanced applications which are not possible using the same materials in their bulk and thin film forms. However, these properties and applications of nanostructures depend upon the method of nanofabrication.

There are several chemical and physical methods that have successfully been used for fabrication of nanostructures of different materials. These chemical and physical methods have several disadvantages like slow deposition rate, contaminants from precursors and catalysts, substrate heating or biasing during deposition, post annealing of deposited material, *etc*. The plasma based methods are often found to be superior to the similar chemical and physical methods.

There are several plasma based nanofabrication methods, such as arc discharge, direct current, radio frequency and magnetron sputtering, pulsed laser deposition, and modified dense plasma focus (DPF) devices, which have been successfully used for the fabrication of a broad range of nanomaterials. Most of these plasma-based methods have certain disadvantages. For example, some of them require substrate heating or biasing, ultrahigh vacuum, or show poor yield, abundant pinholes, some other defects, *etc.* Hot, dense, and extremely non-equilibrium plasmas in modified DPF device overcome most of the above disadvantages and also reduces time for nanofabrication.

The modified DPF device with suitable modification has been used worldwide for the fabrication of nanoparticles and nanostructures of different materials [1,2,3,4,5,6,7,8,9,10,11,12,13,14,15,16,17,18,19,20,21]. In most of these studies, the modification of anode is almost similar but the arrangement for placing the substrate varies. The modified DPF device has been established as a promising tool for nanofabrication by the plasma research group at the University of Delhi [7,8,9,10,11,12,13,14,15,16,17,18,19,20,21]. In particular, several nanostructures of metals [8,9,10,11,12,13], semiconductors [14,15,16,17,18] and insulators [19,20,21] have been produced using the modified DPF device. Apart from this the nanostructures of materials, such as TiC [3], hydroxyapatite (HA) [4], Al/a-C [5], WN_2_ [6], *etc.*, have been fabricated worldwide with the modified DPF device.

The semiconductor materials when fabricated at nanoscale show drastic change in their optical and electronic properties. These changes are mainly due to the quantum confinement effect in the semiconductor nanostructures. There are several semiconductor materials that belong to different groups of the periodic table. The III–V semiconductors are preferred over other semiconductors for microelectronic applications owing to their wide and direct band gap, high electron mobility, low thermal noise, as well as low power consumption.

These properties of III–V semiconductors results in fabrication of devices having wide applications. For example, direct band gap of III–V semiconductors is easily tunable at nanoscale which gives rise to potential applications of the nanostructures in optoelectronic devices operated in broad spectral ranges.

The above properties vary among different nanofabrication methods. The III–V semiconductor nanostructures of materials like gallium nitride (GaN) and gallium arsenide (GaAs) having different morphologies such as nanoparticles [22,23], nanorods [24,25], nanocrystals [26,27], nanopillars [28,29], nanowires [30,31], nanotubes [32,33], nanobelts [34,35], nanoneedles [36,37], *etc.*, have been fabricated by methods such as sol-gel [22], hydride vapor phase epitaxy (HVPE) [38], arc plasma [26], RF sputtering [23,39], inductively coupled plasma (ICP) [28], thermal evaporation [40], electrochemical [41], molecular beam epitaxy (MBE) [42], pulsed laser ablation [43], pulsed electron deposition [44], pulsed laser deposition (PLD) [45], laser-assisted catalytic growth [46], and modified DPF devices [16,17,18].

Most of these methods have limitations, such as slow rates of deposition, contaminants arising out of precursors and catalysts, large power consumption during the heating, and biasing of substrate, as well as the need for ultra-high vacuum and post-deposition annealing. These limitations have been overcome using the modified DPF device for nanoscale synthesis.

The modified DPF device has been successfully used for the fabrication of nanostructures of III–V semiconductors, such as GaN [16] and GaAs [17,18], without any substrate heating/biasing and annealing of deposited material. In particular, GaAs has potential applications in photovoltaic [44], electronic and optoelectronic [17,47,48] devices. Apart from the GaAs nanostructures, the porous GaAs can also be used as a substrate for the growth of other semiconducting materials, such as gallium nitride [49,50]. Moreover, this material produces emission in the visible range which can be used for sensors, light emitting and plasmonic devices. The porous GaAs can also increase the efficiency of the photoelectric and photovoltaic devices, such as solar cells, due to reduced optical losses and other interesting properties.

The formation of porous GaAs has been reported in the literature mainly from the chemical based methods which involve electrolysis and etching to form porous GaAs [49,50,51,52,53,54]. To the best of our knowledge, the formation of porous GaAs using physical or plasma based methods without any etching or electrolysis has not been reported yet. In addition, the fabrication of any porous nanostructures using the modified DPF device has not yet been reported previously.

In this paper, we highlight the applications of modified DPF device for nanoscale synthesis and processing of GaAs on different substrates under different deposition conditions. In particular, we present for the first time fabrication of porous nano-GaAs using the modified DPF device with two shots on glass substrates placed at 4.0 cm distance. Subsequently, we present the characterization results obtained for the as-grown and rapid thermally annealed porous nano-GaAs.

## 2. Results and Discussion

**Figure 1:**
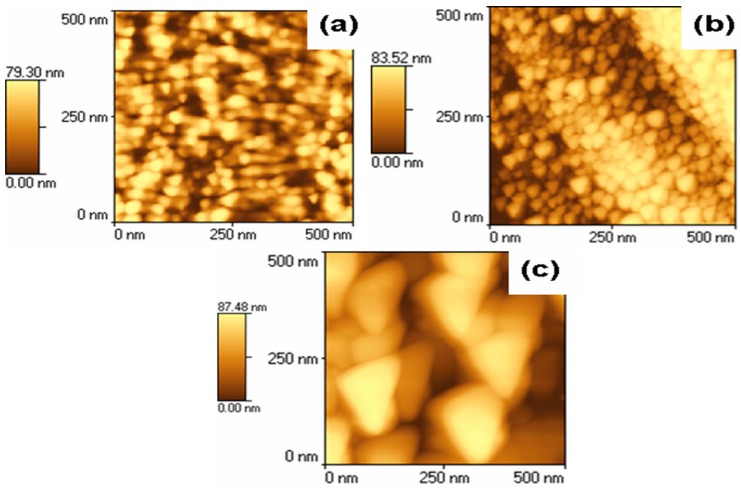
Atomic force microscopy (AFM) images of gallium arsenide (GaAs) nanostructures for (**a**) one, (**b**) two, (**c**) three shots on a silicon substrate placed at a distance of 4.0 cm.

GaAs nanostructures were deposited on silicon and glass substrates using high fluence ions in the modified DPF device. The morphology of GaAs nanostructures deposited on silicon substrate placed at a distance of 4.0 cm indicates the formation of triangular nanostructures mapped by the atomic force microscopy (AFM), as shown in Figure 1. The dimensions of nanostructures produced using 1, 2, and 3 focused DPF shots are found to be in the range of 10–30 nm, 20–50 nm and 40–70 nm, respectively. Therefore, the dimensions of nanostructures increased with the increase in the number of DPF shots.

This may be understood as follows. The material is deposited on the substrate randomly in each shot. In the first shot, the material will deposit on the substrate randomly whereas in the second shot the material is either deposited on as-fabricated nanostructures or on the substrate areas having no deposited material on it. In the third shot the material is deposited by the process similar to that in the second shot, which will results into increased dimensions of the nanostructures.

Figure 1 also shows that the surface density of the nanostructures decreases with increasing the number of shots, which is due to the larger sizes and possible agglomeration of the nanostructures. On the other hand, the morphology of GaAs nanostructures on silicon substrate at 5.0 cm distance shows the formation of nanostructures of arbitrary shape, nanodots and agglomerated nanodots in one, two and three shots with the dimensions of nanostructures in the range of 10–30 nm, 20–40 nm and 40–60 nm, respectively.

**Figure 2:**
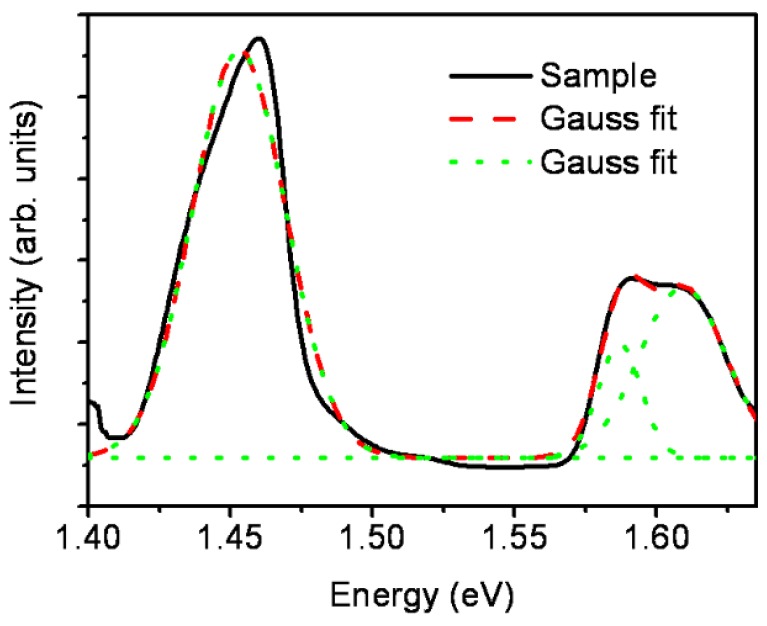
Typical photoluminescence (PL) spectrum of GaAs nanostructures deposited on a silicon substrate placed at a distance of 4.0 cm.

The nanostructures produced on a silicon substrate placed at 4.0 cm and 5.0 cm were further studied for their structural, optical and electrical properties. These properties of GaAs nanodots, deposited with two shots on silicon placed at a 5.0 cm distance, have been reported previously [17]. These results are almost similar for one and three shots cases at a 5.0 cm distance. On the other hand, the PL spectra for triangular GaAs nanostructures shows peaks related to band edge emission and surface recombinations. These peaks are similar to the peaks obtained at a 5.0 cm distance [17]. A typical photoluminescence (PL) spectrum of triangular nanostructures is shown in Figure 2. The PL peak due to surface recombinations is split at about 1.6 eV to show two peaks at 1.59 eV and 1.61 eV. These two peaks are due to surface recombination processes and are in good agreement with the peaks reported by Nayak *et al.* [41].

**Figure 3:**
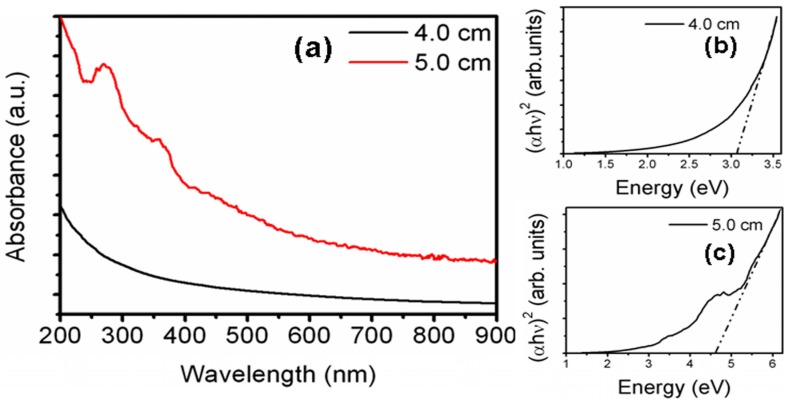
(**a**) Absorption spectra of GaAs nanostructures deposited on silicon substrate placed at a distance of 4.0 cm and 5.0 cm. Tauc plot showing band gap for (**b**) 4.0 cm, (**c**) 5.0 cm distances.

The absorption spectrum of triangular GaAs nanostructures shown in Figure 3a is similar to the spectrum of the nanostructures deposited at a 5.0 cm distance [17]. However, the absorption spectra at 4.0 cm do not possess any excitonic feature as observed at a 5.0 cm distance. Moreover, the band-gap values obtained from Tauc plot at 4.0 cm distance (~3.06 eV) are smaller compared to the 5.0 cm distance (~4.62 eV) as shown in Figure 3b,c, respectively. The electrical properties of the nanostructures deposited on silicon at both 4.0 cm and 5.0 cm distances are identical giving the resistivity values in the range of 7–8 Ω·cm [17].

The GaAs nanodots were also fabricated on glass substrate with two shots at a 5.0 cm distance. The morphological and optical studies of these GaAs nanodots have been reported [18]. The nanodots of 6, 10, and 13 nm sizes were smaller than the Bohr exciton diameter (30 nm) of GaAs. This has resulted in the quantum confinement effect and a clear shift of the band-gap from 1.43 eV for bulk GaAs to 2.88, 2.60, and 2.40 eV for 6, 10, and 13 nm, respectively [18].

The effect of effective mass ratio of electrons and holes has been discussed and it was reported that the deviation between the experimental and theoretical band gap values is not due to approximated spherical shape but it is due to the change in effective mass ratio at nanoscale [18]. On the other hand, if the glass substrates are placed at a distance of 4.0 cm from the top of the anode, then porous nano-GaAs is formed in two shots. The porosity of the nano-GaAs decreases upon rapid thermal annealing of as-deposited (sample A) sample at temperatures of 100 °C (sample B), 200 °C (sample C) and 300 °C (sample D) for 280 ms. The formation of porous nano-GaAs using modified DPF device has been observed for first time and some of the original results on its characterization are presented below. In addition, it is possible to optimize the temperature of annealing for the given pores size but in the present study we investigated the effect of annealing on pores size.

**Figure 4:**
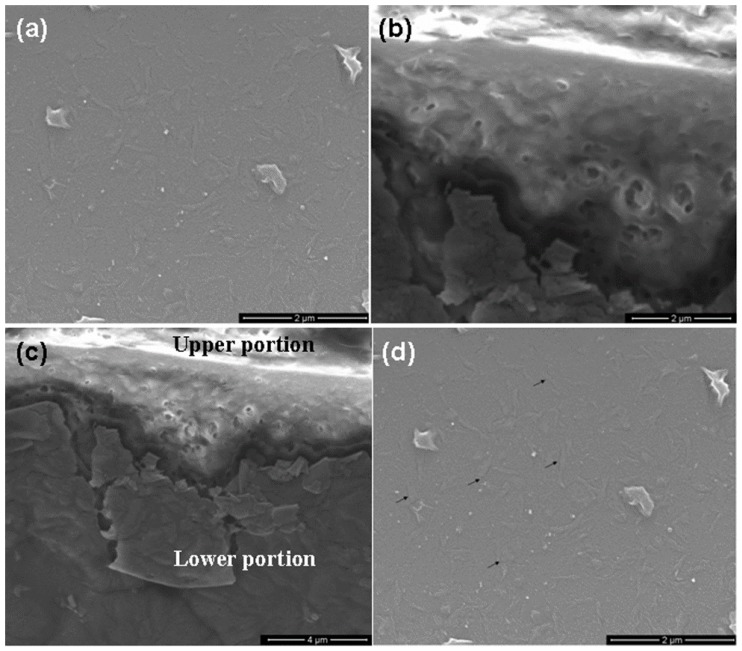
Scanning electron microscopy (SEM) images of as-deposited porous GaAs (sample A). (**a**) surface, (**b**) cross-section view, (**c**) cross-section view showing upper and lower portions, (**d**) pores shown by arrow on surface.

**Figure 5:**
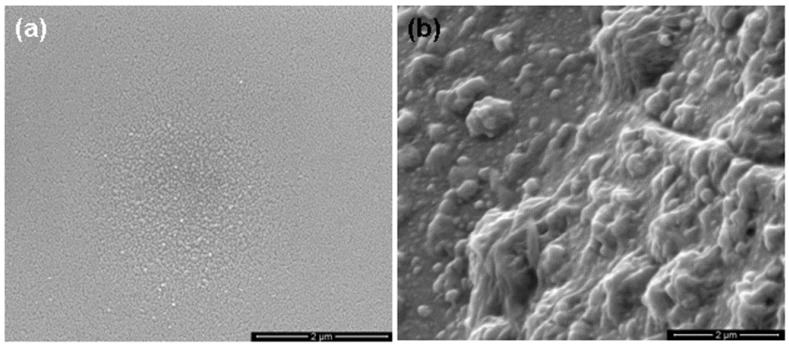
SEM images of 100 °C annealed porous GaAs (sample B). (**a**) surface, (**b**) cross-section view.

**Figure 6:**
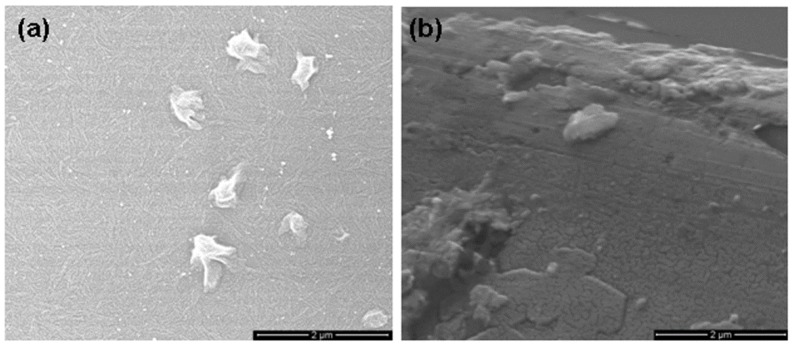
SEM images of 200 °C annealed porous GaAs (sample C). (**a**) surface, (**b**) cross-section view.

Figure 4a, Figure 5a and Figure 6a show the scanning electron microscopy (SEM) images of sample A, B and C, respectively, which indicates the formation of porous GaAs with uniformly distributed pores. The pore sizes have been found for samples A, B, and C from cross-sectional SEM images in Figure 4b, Figure 5b and Figure 6b, respectively. The cross-sectional SEM images show pores that extend from the surface into the sample. The cross-sectional image of large portion of the sample A, showing upper and lower portions is shown in Figure 4c. It is interesting to note that the pores can easily be seen in the surface view of the SEM image. Surface SEM image of sample A, showing pores by arrows is shown in Figure 4d. The average size of pores is found to be 0.2 μm, 0.15 μm and 0.075 μm for sample A, B and C, respectively. It has been found that the average size and the surface density of the pores decrease upon annealing. We have found that the sizes of pores are further decreased upon increasing the annealing temperature to 300 °C for sample D. In addition, we have observed that the size of nanoparticles increases with increasing the annealing temperature and they are of the order of 6 nm, 8 nm, 12 nm and 15 nm for samples A, B, C and D, respectively.

The observed decrease in the average size and surface density of the pores upon annealing can be understood as follows. It is well known that the pores are generally treated as defects in nanostructured films. It is observed in thin films that annealing removes the defects in the material. In the same way nanostructured films processed by DPF device were annealed and pores in these films were drastically reduced by annealing. This is possibly because of the process of rearrangement of GaAs to occupy the unfilled pores or due to agglomeration of nanoparticles outside of the pores caused by the annealing.

**Figure 7:**
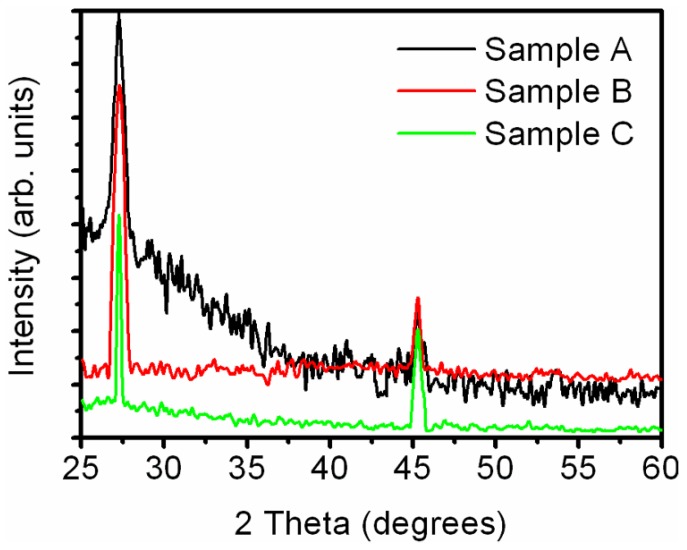
X-ray diffraction (XRD) pattern of as-deposited and rapid thermal annealing (RTA) porous GaAs.

To ascertain the structure of the deposited material, X-ray diffraction (XRD) pattern of as-deposited and annealed samples has been investigated. XRD pattern of as-grown sample A and annealed samples are shown in Figure 7. The XRD pattern of as-grown sample indicates the presence of peaks at 2θ values of 27.3° and 45.3° corresponding to [111] and [220] diffraction planes of zinc blende GaAs (JCPDS File No. 14-450), respectively. The grain size has been calculated using Debye-Scherrer’s Equation:

(1) $$D=\frac{0.9\lambda }{\beta \cos\theta }$$

where *D* is the average grain size, λ is the wavelength, β is the full width at half maximum (FWHM) of the peak in radians, and θ is the angle of diffraction corresponding to the peak. The grain size is found to be ~10 nm and ~12 nm for [111] and [220] planes, respectively. Thus, the XRD results confirm the deposition of GaAs having nano-dimensional grains on the glass substrate. The XRD of the annealed samples has shown the peaks at the same position *viz*. 27.3° and 45.3° corresponding to [111] and [220] diffraction planes of GaAs, respectively. The grain sizes for the annealed samples are also of the same order as those obtained for the as-grown samples.

**Figure 8:**
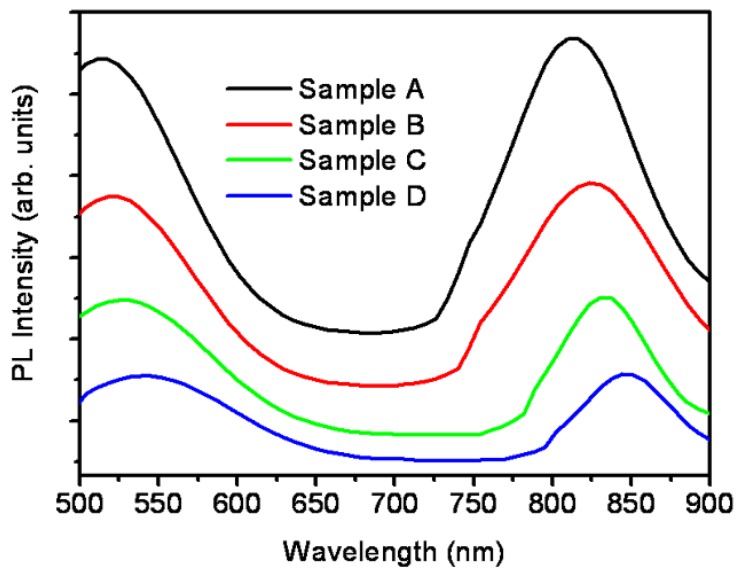
PL spectra of as-deposited and RTA porous GaAs.

The PL spectra of as-grown porous GaAs reveal the emission peaks at 511 nm and 817 nm. This emission suggests possible applications of the porous GaAs in sensors and light emitting devices. The changes in PL spectra with rapid thermal annealing were further studied. The PL spectra of the as-deposited porous GaAs along with the annealed samples are shown in Figure 8. For sample B which is annealed at 100 °C the above peaks are red-shifted to 518 nm and 824 nm. A similar red shift was observed when samples C and D were annealed further at 200 °C and 300 °C. In particular, the peaks were further red shifted to 525 nm and 831 nm for sample C and 538 nm and 844 nm for sample D.

The observed red shift in PL peaks upon annealing is likely due to a decrease in the surface density of the pores. The peaks observed in infra-red region at 817, 824, 831, and 844 nm are band-edge emission of GaAs having blue shift of ~50, 43, 36, and 23 nm, respectively, from the 867 nm peak of infrared emission originating from bulk GaAs [55].

The PL peaks observed in the visible region at 511, 518, 525, and 538 nm represent green emission from porous GaAs [49,53,56,57,58]. In literature, the green emission has been observed in PL spectra of porous GaAs due to the presence of nanocrystallites having size of the order of 7 nm [49].

We have also have estimated the size of nanocrystallites from the green emission peak using the effective mass theory. Assuming the infinite potential barriers, the energy gap *E* of GaAs nanocrystallites confined in three-dimensions should vary as [55],

(2) $$E=E_{g}+\frac{h^{2}}{2d^{2}}\left(\frac{1}{m_{e}^{*}}+\frac{1}{m_{h}^{*}}\right)$$

where *E_g_* is the energy bandgap of bulk GaAs, *d* is the diameter of nanocrystallites, while m_e_^*^ and m_h_^*^ are the effective masses of the electrons and holes, respectively. At 300 K, *E_g_* = 1.425 eV, m_e_^*^ = 0.063*m*_0_ and m_h_^*^ = 0.53*m*_0_ [55], where *m*_0_ is the electron mass in vacuum. The green PL peaks observed at 511, 518, 525, and 538 nm corresponds to the energies ~2.426, 2.394, 2.362, and 2.304 eV, respectively. From these data the value of *d* was estimated to be ~5 nm in all the four cases. Thus, the porous GaAs fabricated through the present technique possesses nanocrystallites having sizes of the order of 5 nm which give rise to green emission peak in PL spectra.

**Figure 9:**
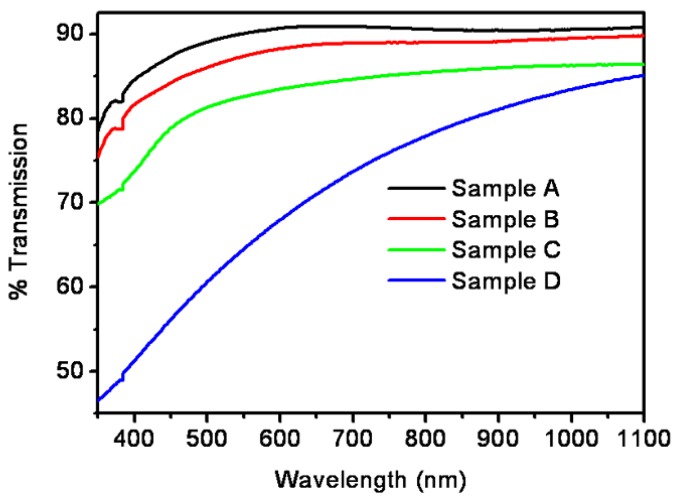
Transmission spectra of as-deposited and RTA porous GaAs.

Transmission measurements give optical losses in porous GaAs. It is observed in the present study that there is a decrease in the percentage of transmission from 78% to 45% with increasing the annealing temperature in the shorter-wavelength range from 350 to 900 nm (Figure 9). On the other hand, in the longer-wavelength range from 900 to 1100 nm the percentage transmission is in the range of 80%–90% for all the four samples. This variation in the transmission spectra is due to the defect-related states present in the porous nano-GaAs. The increase in the transmission of GaAs in the infra-red region due to defect states has also been reported in the literature [59,60]. The high percentage transmission of the deposited and subsequently subjected to rapid thermal annealing (RTA) GaAs can find potential applications in transmission-type photocathodes [61]. The RTA process can, thus, be used for suppression the pore defects and for tailoring the pore, and consequently, the nanoparticle sizes.

The fabricated porous nano-GaAs has emission in visible range which suggests that the fabricated porous GaAs can be easily used in making nano-optoelectronic devices in the visible range. Moreover, the porous GaAs produced in the present experiment is the first time achievement by the physical based plasma method. Thus, the obtained porous nano-GaAs possess all qualities required for device fabrication. In addition, due to use of plasma based physical method the possibility of presence of contaminations is low which subsequently render the porous nano-GaAs more suitable for device fabrication. The porous GaAs fabricated earlier by Salehi and Kalantari [62] has already been used for making CO and NO gas sensors.

## 3. Experimental Section

**Figure 10:**
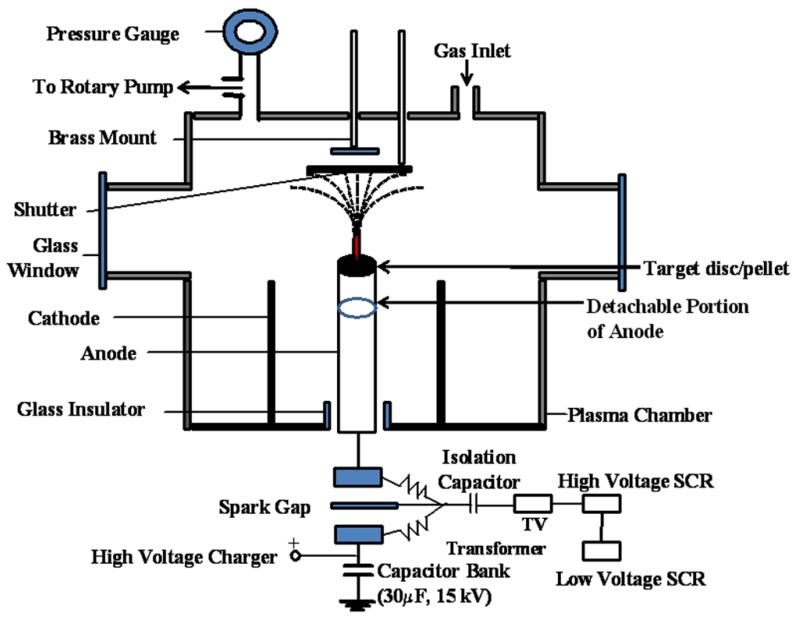
Schematic of modified dense plasma focus device.

A schematic of the DPF device with the modified anode and other modification is shown in Figure 10. The DPF device used is a 3.3 kJ Mather type device [63], powered by 30 µF, 15 kV energy storage capacitor. The anode of the DPF device has been modified such that a disc/pellet of a target material can be fixed at the top of it. The nanofabrication of different materials has been done on different substrates in the modified DPF device through high fluence of ions of the deposited material. The cleaned substrates are mounted on a perspex holder having a threaded hole at the center for screwing in the vertically moveable brass rod. The whole assembly is inserted from the top of the plasma chamber and its distance from the anode top is adjusted from outside the plasma chamber using a brass rod. The substrates are kept at room temperature. An aluminum (Al) shutter is also introduced from the top of the plasma chamber using another moveable brass rod. The shutter is placed in between the anode top and the substrates in order to protect the substrates from the impact of ions produced by unfocused plasmas. The chamber is evacuated using a rotary pump and then argon gas is introduced into the chamber as a working gas. The plasma chamber is flushed with argon gas several times to maintain the inert atmosphere. It has been established earlier [64] that an optimum argon gas pressure of 80 Pa is required for obtaining focused plasmas.

In our experiments, we have also observed that a good focus is obtained at optimum argon gas pressure of 80 Pa in the chamber. The generation of the focused plasmas is observed as a sharp peak in the voltage signal which is recorded on a digital storage oscilloscope (Tektronix TDS 1002, Bangalore, India). The plasma has a density of the order of 10^26^ m^−3^, temperature 1–2 keV and only lasts ~100 ns in the focus phase. At this stage, approximately 10^17^ ions (argon ions and ions of the deposited material) per shot are produced. The focused plasma ionizes the material disc/pellet surface and ions of the ablated material move vertically upwards in a fountain-like shape in the post-focus phase and then get deposited on the substrates. More discussions of the unique features of DPF devices and other methods of energetic ion deposition can be found elsewhere [65]. We have used one, two and three focused shots for nanofabrication of III–V semiconductors. The substrates were placed at 4.0 cm and 5.0 cm distance from the top of the anode.

## 4. Conclusions

The advantages of the modified DPF device are highlighted over the other deposition methods. In particular, this paper discussed the deposition of III–V semiconductor nanostructures using the high fluence ions generated in the modified DPF device. The brief results of the GaN and GaAs nanostructures fabricated using the modified DPF device have been discussed. The details of the first time fabrication of the porous nano-GaAs using modified DPF device are presented. It is found that RTA technique can be used to tailor the pore sizes in the porous nano-GaAs. The presence of nanograins in the porous GaAs samples has been confirmed from the XRD pattern. Furthermore, porous GaAs show strong PL emission in visible and infra-red regions along with high transmission. The obtained change in optical properties of porous GaAs on annealing is consistent with the changes observed in morphology. The porous GaAs fabricated and subsequently annealed in this work is promising for applications in visible optoelectronic devices and transmission type photocathodes.
